# Epidemiology study of HBV genotypes and antiviral drug resistance in multi-ethnic regions from Western China

**DOI:** 10.1038/srep17413

**Published:** 2015-11-27

**Authors:** Qi Zhang, Yun Liao, Jie Chen, Bei Cai, Zhenzhen Su, Binwu Ying, Xiaojun Lu, Chuanmin Tao, Lanlan Wang

**Affiliations:** 1 Department of Laboratory Medicine, West China Hospital, Sichuan University, No. 37 Guoxue Alley, Wuhou district, Chengdu 610041, People’s Republic of China

## Abstract

Hepatitis B virus (HBV) infection is a critical global health issue and moderately epidemic in Western China, but HBV molecular epidemiology characteristics are still limited. We conducted this study to investigate HBV genotypes and antiviral resistant mutations in this multi-ethnic area. A total of 1316 HBV patients were recruited from four ethnic groups from 2011 to 2013. Genotypes and resistant mutations were determined by Sanger sequencing. Four genotypes (B, C, D and C/D) were identified. Genotype B and C were common in Han population, while genotype D was predominant in Uygurs. Genotype C was the major genotype in both Tibetans and Yis, and recombinant C/D was found in Tibetans only. Lamivudine resistance was common in all populations, especially in Hans with prevalence of 42.8%. Entecavir resistance was barely observed regardless of ethnicity. Genotype C isolates had higher rates of rtA181T/V than genotype B (13.5% vs. 5.1%, P < 0.001), in accordance with higher prevalence of resistance to adefovir (20.0% vs. 9.5%, P < 0.001). While incidence of resistant mutations to other drugs and clinical factors showed no difference among different genotypes. HBV genotypes and resistance-conferring mutations had different geographic and demographic distributions in Western China, which provided molecular epidemiology data for clinical management.

Hepatitis B is one of the major public health priorities with more than 350 million chronic hepatitis B virus (HBV) carriers over the world and causing 686, 000 deaths per year^1^,^2^. China used to be a highly endemic country of HBV (prevalence ≥ 8%), and once a nationwide HBV investigation showed that hepatitis B surface antigen (HBsAg) carrier rate in general population was 9.75%^3^. Since the initiation of universal HBV vaccination in all newborns from 2002, the HBsAg prevalence of the whole population in China has decreased dramatically to 7.18% in 2006, with a little higher rate of 7.9% in Southwest area^4^.

HBV has an incomplete circular double-stranded DNA genome. According to more than 8% genetic variability in the full-length nucleotide sequence or 4%–8% divergence in the S gene, at least 10 HBV genotypes (A to J) and their subtypes have been defined^5^,^6^,^7^. Genotype A is mainly prevalent in sub-Saharan Africa, Northern Europe and Western Africa. Genotype B and C are common in Asia. Genotype D prevails in Africa, Europe, the Mediterranean region and India. Genotype E was prevalent in West and Central Africa previously and now could be found in sporadic cases of immigrants or tourists in Europe, Turkey, Northern India and Latin America. Genotypes F to J are less epidemic and usually have their own specific distributions^8^,^9^. HBV Genotypes A-H are approved genotypes and their distributions have been well characterized, while genotypes I and J are tentative genotypes defined according to their genome divergencies^7^,^10^.

In China, genotype B is predominant in Northern areas, and genotype C is more common in Southern areas^11^,^12^. Besides geographic distributions, HBV genotypes also have demographic differences. It has been reported that high prevalence of C/D recombinant is identified in Tibetans in Tibet and Qinghai, and the recombinant also could be found in Yunnan Province sporadically^13^,^14^,^15^. Genotype D is mainly found in Xinjiang Uygurs^14^,^16^. These two HBV genotypes are both rare in Han population. Studies also indicated that different HBV genotypes underlie the varying clinical and biological characteristics of chronic hepatitis B (CHB) patients to some extent. Genotype A and C have a higher tendency of chronicity in natural history, while genotype D and C have a higher risk of development to cirrhosis and hepatocellular carcinoma (HCC)^8^. In hepatitis B e antigen (HBeAg) positive patients, genotype A and B patients are more susceptive to standard interferon α (IFN-α) therapy, and have a higher sustained response rate than genotype D and C, respectively^17^,^18^. Genotype A patients could present higher HBeAg and hepatitis B surface antigen (HBsAg) clearance than genotype B, C and D when treated with pegylated IFN-α, regardless of HBeAg status^19^.

Currently, seven antiviral agents are approved for management of CHB infection including two formulations of IFN-α (conventional and pegylated), and five nucleos(t)ide analogues (NAs), namely lamivudine (LMV), adefovir (ADV), telbivudine (LdT), entecavir (ETV) and tenofovir (TDF)^20^,^21^. Although IFN-α has directly antiviral and immunomodulatory activity, the majority of CHB patients are treated with NAs only and interferon therapy is applied to a few selected patients in consideration of side-effects^22^. NAs suppress HBV replication mainly by restraining the reverse transcription of the pregenomic RNA into DNA, so the amino acid substitutions in HBV reverse transcriptase (RT) will lead to NAs resistance, which is a tremendous challenge in the management of CHB. LMV is the first approved NA and utilized widely, but it also has the highest rate of antiviral resistance from 14–32% after 1 year treatment to 60–70% after 5 years in clinical trials with CHB patients^23^. ADV and LdT have high efficacy of inhibiting HBV and moderate incidences of drug resistance, while ETV and TDF have been shown to have a high barrier to resistance with rates of less than 1.5% after 5 years in NAs naïve CHB patients^24^. Disparate antiviral resistant mutations will result in reduced susceptibility to single or multiple antiviral agents. As a multi-drug resistant mutation, rtA181T/V has cross-resistance to LMV, LdT and TDF as well. Combination of rtM204V/I, rtL180M plus one of the following mutations: rtT184S/C/I/A, rtS202G/C/I or rtM250V links to ETV resistance. Emergence of such antiviral drug resistant mutations contradicts the benefits of antiviral therapy and even triggers hepatitis flares, liver failure and death for the uncontrollable HBV replication and cascade of liver inflammation.

As depicted before, CHB patients carrying different HBV genotypes could have diverse outcomes. The response to NAs and prevalence of NAs-induced resistance between different HBV genotypes also evoke much altercation. Two large-scale clinical studies from China found that rtM204I with or without rtL180V was more frequent in genotype C than genotype B^25^,^26^. While, Spanish scholars reported comparable incidence of LMV-resistance and outcome in Caucasian CHB patients between genotype A and D^27^. Some other studies also showed no statistical difference in response to LMV and LdT among CHB patients with various genotypes^28^,^29^,^30^. Although there are no definite differences in response to nucleos(t)ide analogues between HBV genotypes, our previous study showed that genotype C had a higher incidence of natural antiviral-resistant mutation than genotype B and D, which could lead to poorer response and outcome^31^. And whether HBV genotypes correlated to different drug resistance rates needs to be further investigated.

Western China is a multi-ethnic area, including Han residing in most provinces, Tibetan mainly in Tibet and Sichuan province, Uygur in Xinjiang, Yi and Miao in Yunnan province, and so on. These diverse populations have distinct lifestyles, socio-economy, culture and genetic backgrounds. HBV prevalence in Western China is a bit higher than that in Eastern China, however, data of HBV molecular epidemiology from Western China is rather scarce. Up to now, there is very limited information about HBV genetic discrepancy, including genotype distribution, HBV-DNA load and prevalence of antiviral resistant mutations between different ethnic minorities in Western China. Therefore, we conducted a retrospective study to investigate HBV genotypes and drug resistant mutations in Sichuan, Tibet, Xinjiang and Qinghai to provide detailed molecular epidemiology information of HBV for evaluating the potential future disease burdens and the management strategies.

## Results

### Demographic and Clinical Characteristics of study patients

A total of 1316 HBV HBsAg carriers were recruited from five hospitals in four provinces, and HBV reverse transcriptase fragment was amplified successfully from 1221 samples consist of 657 Hans, 472 Tibetans, 46 Uygurs and 46 Yis. The median age of total patients was 36 (interquartile range: 27, 48) years, and 74.53% of them were male. The median serum HBV DNA was 6.27 (interquartile range: 4.68, 7.05) log10 copies/ml. All of the patients were positive for HBsAg and antibody to hepatitis B core antigen (anti-HBc), and 97.38% of them were HBeAg positive (Table 1).

### Geographic and demographic distribution of HBV genotype in Western China

**Figure 1:**
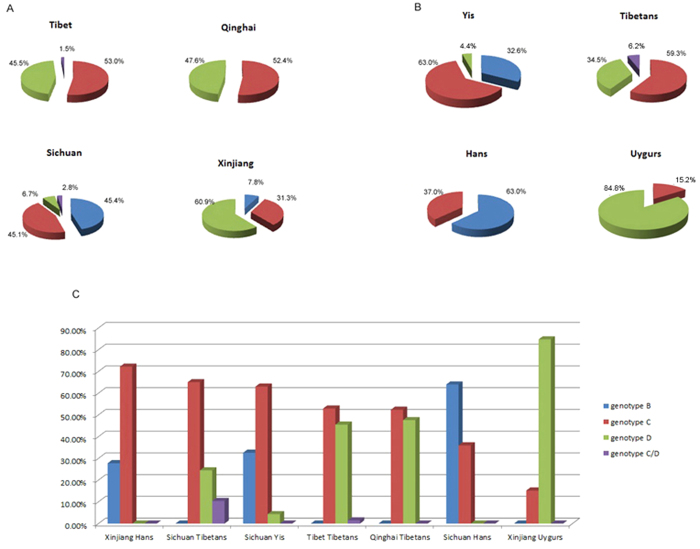
Geographic and demographic distribution of HBV genotypes in Western China. (**A**) Different HBV genotype distributions in four regions from Western China. (**B**) Different HBV genotype distributions in four ethnic groups from Western China. (**C**) Each ethnic group from different regions illustrates special HBV genotypes distribution from Western China. The pie-chart and columns represent the prevalence of HBV genotypes, and different colours represent different genotypes: blue-genotype B, red-genotype C, green-genotype D, and purple- recombinant C/D.

In total, four HBV genotypes (B, C, D and C/D) were identified in the 1221 samples. Among them, 429 (35.1%) patients were infected with genotype B, 559 (45.8%) with genotype C, 204 (16.7%) with genotype D, and 29 (2.4%) with recombinant C/D. The geographic distribution of HBV genotypes in five provinces from Western China varied extraordinarily. Genotype B and C were common in Sichuan with comparative prevalence of 45.4% and 45.1%, respectively. Genotype D was predominant in Xinjiang, followed by genotype C and B. In Tibet and Qinghai, the main prevalent HBV isolates were genotype C and D, with a little higher rate of genotype C than genotype D ( genotype C and D in Tibet: 53.0% vs. 45.5%, and in Qinghai: 52.4% vs. 47.6%). Recombinant C/D was only found in Sichuan and Tibet (Fig. 1A).

In the subgroup analysis stratified by ethnic population, significant differences of HBV genotype distribution were observed among Hans, Tibetans, Uygurs and Yis (Fig. 1B). The main genotypes in Hans were B and C with a bit difference between Sichuan Hans and Xinjiang Hans (Fig. 1C). Most Sichuan Hans (64.0%) were infected with HBV genotype B, while genotype C was more common in Xinjiang Hans (72.7%). The predominant genotypes in Tibetans were C and D, partly C/D recombinant (6.2%), and genotype C was slightly more than genotype D (59.3% vs. 34.5%). Tibetans from different areas (Tibet, Sichuan and Qinghai) had similar genotype distributions, with genotype C predominant and followed by genotype D. Uygurs in Xinjiang showed a unique high prevalence of genotype D (84.8%), which was different from Hans and Tibetans. The predominant genotype in Yis was genotype C (63.0%), and smaller patients were infected with genotype B and D (32.6% and 4.4%, respectively).

**Figure 2:**
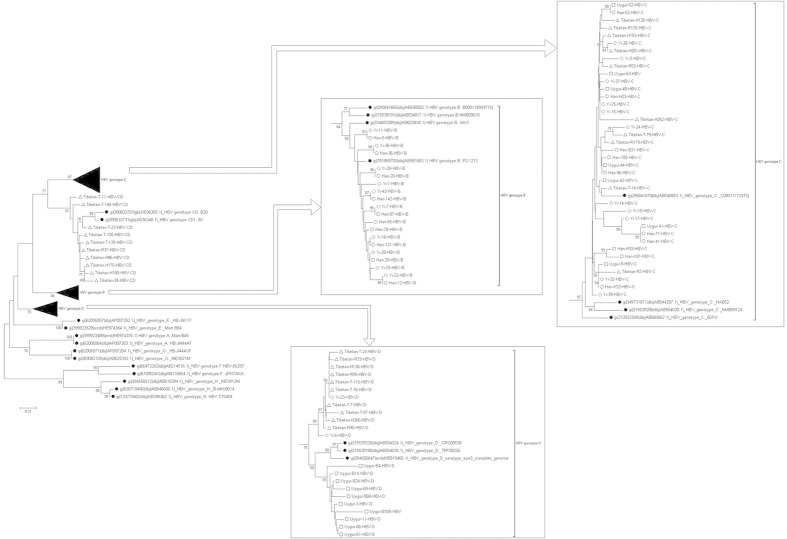
Phylogenetic analysis of HBV RT region from samples in Western China and 24 reference sequences from genotype A-H available at GenBank. The tree was generated with MEGA 5.0, using the Neighbor-Joining method and 1000 bootstrap replicates. The support values for the branches are indicated at the nodes, and HBV genotypes are listed on the right. The HBV isolates from Han are marked with white dots, HBV isolates from Tibetan with white triangles, HBV isolates from Uygur with white boxes, and HBV isolates from Yi with white diamond. HBV reference sequences retrieved from GenBank are marked with black dots and labelled with the accession number.

**Figure 3:**
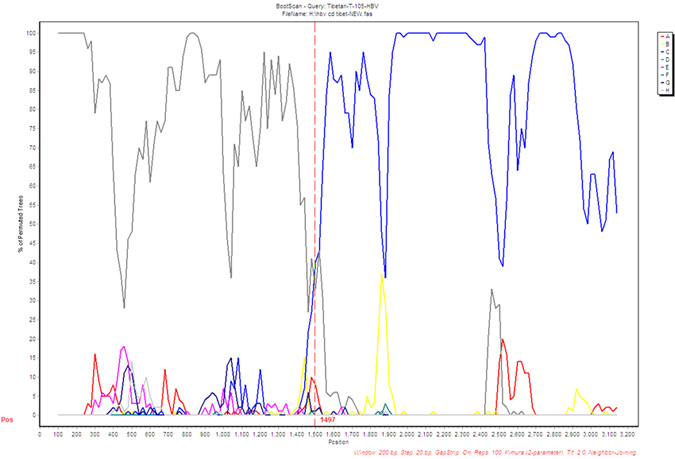
SimPlot bootstrap analysis on complete genome sequence of the recombinant genotype. The recombinant genotype was verified through SimPlot bootstrap analysis. Sequence from nucleotide 1 to 1497 showed high similarity with genotype D and the remaining sequence showed high similarity with genotype C.

Then we conducted the phylogenetic analysis to confirm the genotyping method. We randomly selected 10 of the HBV isolates in each genotype from all of the ethnic groups by using the random number table to do the phylogenetic tree analysis. If the number of HBV isolates in the group was less than 10, we chose all of them. Figure 2 showed that our genotyping method had correctly determined the HBV genotypes. The genotype D isolates from Tibetan and Uygurs could be classified into different subgenotypes based on their bootstrap values, while genotype B and C from different ethnic groups have close phylogenetic relationship. For the C/D recombinant, we further analyzed their complete genome sequences to verify the existence of recombinant strains by performing SimPlot bootstrap analysis. Figure 3 showed the result of one C/D recombinant isolate for example, and the breakpoint of the recombinant was at 1497 nucleotide.

### Genotypes and clinical features in Han population

For further analysis of the association between HBV genotypes and clinical characteristics, 639 Han HBV subjects in Sichuan were included to compare their demographic information and clinical laboratory markers (Supplementary Table S1). The distribution of HBV genotype B and C between males and females in Sichuan Hans were not distinct significantly, but patients infected with genotype C were older than patients with genotype B (mean ± standard deviation: 37.34 ± 10.33 vs. 34.90 ± 10.22, P = 0.004). The serum HBV DNA load made no difference between genotype B and C (P = 0.25), and HBV genotypes also had no influence on liver function biomarkers, such as alanine aminotransferase (ALT), aspartate aminotransferase (AST), alkaline phosphatase (ALP), and gamma glutamyl transpeptidase (GGT), etc (P > 0.05).

### Geographic and demographic distribution of HBV antiviral resistant rate in Western China

**Figure 4:**
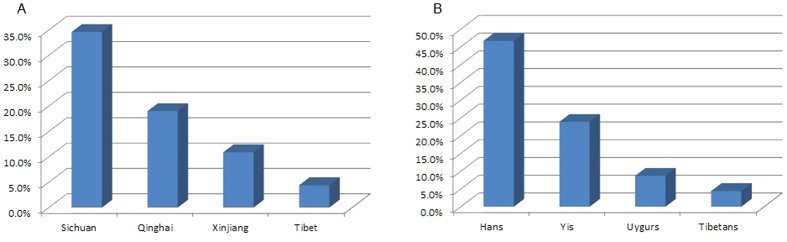
Geographic and demographic distribution of overall HBV resistance to antiviral agents in Western China. (**A**) Different rates of HBV resistance to antiviral agents in four geographic regions from Western China. (**B**) Different rates of HBV resistance to antiviral agents in four ethnic groups from Western China. The columns represent the overall rates of HBV resistance to antiviral drugs.

HBV antiviral resistant mutations were determined for all of the 1221 patients, and analysis was carried out to evaluate four widely used NAs in China, namely LMV, LdT, ADV and ETV. The overall resistant rate showed variability among different areas. Sichuan had the highest prevalence of antiviral drug resistance of 34.6%, followed by Qinghai and Xinjiang with the pooled prevalence of 19.0% and 10.9% respectively, and Tibet with the lowest resistant rate of 4.4%. In ethnic-specific subanalysis, the resistant rate in Hans reached up to 46.7%, compared with 23.9% in Yis, and less than 10% resistant rate was observed both in Tibetans and Uygurs (Fig. 4A).

In Han patients, resistant rate to LMV ranked the first with the prevalence of 42.8%, followed by 37.0% resistance to LdT. However, Han patients in different areas had different distributions of resistance to four antiviral agents. HBV isolates in Sichuan Hans showed a high resistant rate to all of the NAs, while in Xinjiang Hans, relatively high resistance to LMV (16.7%) was only observed. As to Tibetans and Uygurs, resistance to LMV was also the major source of antiviral resistant rate with the prevalence of 4.2% and 8.7% respectively, and resistance to ADV and ETV were seldom found, except Qinghai Tibetans with a resistant rate of 4.8% to ADV. In Yi patients, the predominant drug resistance occurred in LMV and LdT (both of 17.4%) compared with 6.5% in ADV, and no resistance to ETV was observed as well (Fig. 4B, Supplementary Table S2).

### HBV genotypes and antiviral resistant mutations in Han population

Based on 639 Han patients in Sichuan Province, the correlation between HBV genotypes and antiviral drug resistance were estimated. Patients infected with genotype C had a much higher ADV-resistant rate than patients carrying genotype B (20.0% vs. 9.5%, P < 0.001). However, no difference was found when comparing the resistant rate of LMV, LdT, ETV and the total resistant incidence between genotype B and C (Supplementary Table S3).

Next, we took a further step to study the percentage of mutant sites in different HBV genotype strains in these 639 Han patients to comprehend the molecular mechanism of antiviral resistance. As shown in Table 2, ADV-associated mutation rtA181V/T/S demonstrated a remarkable higher prevalence in genotype C than genotype B (13.5% vs. 5.1%, P < 0.001), and so did other mutations (rtN/H238T P < 0.001, rtP237H P = 0.002, and rtV214A P = 0.006). Nevertheless, LMV-associated mutation rtM204V/I and rtL180M, LdT-associated mutation rtM204I, and ETV-associated mutations rtT184A/I/S, rtS202G and rtM250L didn’t differ significantly between genotype B and C (P > 0.05).

In addition, subgroup analysis by NAs was performed in Sichuan Han population to evaluate the effect of HBV genotypes on resistant mutation rate. In patients resistant to LMV and LdT, mutation rtM204V and rtM204I showed no difference between genotypes, while mutant rtL180M had a higher frequency in genotype C isolates than genotype B isolates in both subgroups (54.7% vs. 36.1%, P = 0.002 for LMV-resistant patients; and 63.7% vs. 43.5%, P < 0.001 for LdT-resistant patients) (Supplementary Table S4 and S5). Interestingly, cross-resistant mutation rate of rtA181V/T/S was higher in patients harboring genotype C than genotype B in LMV-resistant patients (29.3% vs. 10.5%, P < 0.001), but not in patients resistant to ADV (39.1% vs. 53.9%, P = 0.175). Besides, another ADV-associated resistant mutation rtN236T had a higher prevalence in genotype B than genotype C in ADV-resistant patients (66.7% vs. 13.0%, P < 0.001) (Supplementary Table S6). In ETV-resistant patients, no difference of mutation rates between genotype B and C was observed (Supplementary Table S7).

Besides the different distribution of single mutant between genotype B and C, we further analyzed the correlation of certain mutation models and HBV genotypes (Table 3). The most common drug resistant mutation model was dual mutation rtL180M+rtM204V, followed by rtL180M+rtM204I. The frequency of LMV-associated mutation models, like rtL180M+rtM204V and rtL180M+rtM204V+rtV173L, showed no statistical difference, except rtL180M+rtM204I, the frequency of which was higher in genotype C than genotype B (10.7% vs. 4.2%, P = 0.026). The rate of other drug-associated resistant mutation models, such as rtA181T+rtN236T for ADV, rtL180M+rtM204V+rtS202G for ETV, and rtL180M+rtM204V+rtT184L for ETV, did not differ between genotypes as well.

## Discussion

In this study, we analyzed the geographic and demographic distribution of HBV genotypes and the prevalence of antiviral resistant mutations of 1221 HBV isolates from four ethnic groups in Western China. We determined four HBV genotypes in all, namely genotypes B, C, D and recombinant C/D, and each ethnic population had different major genotypes. LMV-resistance was common in all populations, and ETV resistance was hardly observed. ADV and LdT resistance was more predominant in Han and Yi groups than Uygurs and Tibetans. Patients infected with genotype C had a higher rate of mutant rtA181T/V and resistance to ADV than genotype B, while the incidence of resistant mutations to other NAs and clinical factors had no difference statistically among different genotypes.

Obviously, we found that the predominant genotypes in Han population were genotype B and C, although the distribution frequency varied among different geographic regions. Genotype C was dominant in Xinjiang Hans, while genotype B predominated in Sichuan Hans, which was in accordance with previous studies about HBV genotypes in Northern and Southern China^16^,^32^. In Tibetans, genotype C was the most epidemic genotype, ensued by genotype D. Noteworthy, we found 6.2% C/D recombinant in Tibetans, which was quite different from the other ethnicities. However, several studies showing the frequency of C/D recombinant in Tibetans was as high as 41.2%–96.1%^14^,^33^. This inconsistency may be owing to the differences in the selection of subjects, numbers of included samples, quantity of tested samples and the method of genotype determination. In addition, Shen *et al.*^33^ reported five Han patients from Southwestern China infected with genotype C/D4. This result has sparked our great concern, since genotype D is rarely found in Han population, and therefore C/D4 recombinant seems to be a little bit inappropriate when observed in Hans. Thus more studies are warranted.

Consistent with previous studies, LMV-resistance was the most common resistance in Western China, with the highest resistant rate of 34.6% in Sichuan Province. In the subgroup analysis of ethnicity, LMV-resistance was the most common in Han population with the prevalence as high as 42.8%. Currently, conflicting results exist in whether HBV genotypes have an impact on LMV resistance. Damerow *et al.*^34^ revealed that rtM204V was selected more frequently in genotype A patients compared to non-genotype A patients. While another two studies pointed out that the development of LMV resistance did not differ among HBV genotypes^30^,^35^. In this study, we did not find any statistical difference in the frequency of rtM204V and rtM204I between genotype B and C (P = 0.119, P = 0.161, respectively) in LMV-resistant patients as well. However, when analyzing rtL180M, a secondary mutation, genotype C showed a higher incidence than genotype B (54.7% vs. 36.1%, P = 0.002), suggesting stronger replication fitness for genotype C virus and more potent resistance to LMV^24^. Another L-Nucleoside, LdT, shared some similar amino acid substitution in polymerase with LMV, namely rtM204I and rtL180M, which could increase the risk of cross-resistance. Our results showed that Sichuan Han population had the highest prevalence of LdT-resistance by 38.1%, followed by Sichuan Yi population with 17.4% resistance rate, but Tibetans from Sichuan and Tibet neither had LdT resistance, which presented remarkable ethnicity multiplicities. In 244 LdT-resistant patients from Sichuan Han population, HBV genotypes seemed not to be an important factor for the occurrence of antiviral resistance, with comparable prevalence of mutation sites rtM204I in genotype B and C (62.8% vs. 54.9%, P = 0.229). But mutant rtL180M was more common in genotype C than genotype B again (63.7% vs. 40.5%, P < 0.001), which was supported by the higher rate of dual mutation rtL180M+rtM204I in genotype C. Therefore, we suggested that LMV monotherapy was not appropriate to CHB patients in Han population from Western China in consideration of such a high incidence of LMV-resistance, and antiviral agents with high genetic barrier should replace LMV to avoid potential treatment failure.

HBV mutation rtA181T/V and rtN236T could lead to drug resistance of ADV, and the influence of HBV genotypes on the resistance to ADV has not been clarified. Yuen *et al.*^36^ analyzed HBV sequences from 1236 CHB patients and found significantly higher frequency of rtA181T in HBV genotypes C isolates compared to genotypes D and A isolates (63.8% vs. 12.8% vs. 0%, P < 0.05), while HBV genotype D strains had a higher rate of a combination of rtA181T/V and rtN236T than genotype B and C (53.9% vs. 17.6% vs. 23.5%, P < 0.05). In our study, ADV-resistance was found in Sichuan Han population, Qinghai Tibetans and Sichuan Yis with the prevalence range from 4.8% to 13.4%. Xinjiang Uygurs and Tibet Tibetans showed no incidence of rtA181T/V and rtN236T. Since genotype B and C were found predominant in Sichuan Han population, while genotype D major in Uygurs and Tibetans, the different geographic distributions of HBV genotypes may influence the prevalence of ADV resistance. The total frequency of rtA181T was higher in HBV genotype C isolates than genotype B isolates in Sichuan Hans (13.5% vs. 5.1%, P < 0.001), which could confer the higher ADV-resistance rate in genotype C infected patients (prevalence: 20.0% vs. 9.5%, P < 0.001). It was noteworthy that ADV-associated mutation rtA181T was considered as a cross-resistant mutation, which could induce resistance to LMV and LdT in patients never exposed to these antiviral drugs. Therefore when the cross-resistant mutations occur, clinicians should be cautious and consider the poor response to NAs before any antiviral drug is prescribed.

ETV is a potent antiviral drug with higher genetic barrier and was recommended to be used as the first line agent in naïve CHB patients. The occurrence of resistance to ETV in drug-naive patients is rather rare during the first year and remains low even after more than 6 years of treatment (approximately 1%)^37^,^38^. But in patients who failed the LMV treatment and switched to ETV therapy, the frequency of virological breakthrough would rise to 50% after 5 years treatment^38^,^39^,^40^. In this study, only 4.2% Han patients in Sichuan showed resistance to ETV, while in other minorities or regions ETV-resistance was scarcely observed. Therefore, HBV strains from Western China were still more susceptive to ETV than other NAs, and ETV was strongly recommended to be used for untreated patients to control the development of resistance.

As is known to all, NAs-experienced HBV patients have a risk of developing drug resistant mutations. In addition, the occurrence of spontaneous resistant mutations in treatment-naïve patients was reported over the world. In China, the incidence of natural resistant mutations varied greatly among different areas. Yang *et al.* found 3.83% (37/966) untreated CHB patients and asymptomatic carriers harboring natural YMDD mutations in a city of Sichuan Province in Western China, and genotype C strains had a higher rate of spontaneous YMDD mutations than genotype B^41^. While a study including HBV patients in Beijing from Northern China reported the rate of natural YMDD mutations was 1.78% (15/845)^42^. And a small-scale research on naïve HBV patients in Shanghai from Eastern China found no well-characterized primary resistant mutations^43^.

This cross-sectional study provided an integrated landscape of HBV genotypes and resistant mutations in West China, however, there were some limitations in our study. First, clinical features of CHB patients from Tibet, Xinjiang and Qinghai were not available and the analysis of HBV genotypes and clinical characteristics was only taken in Sichuan Han population. Second, we determined HBV genotypes and resistant mutations by direct sequencing, which has a lower sensibility than Ultra-deep pyrosequencing^44^. Third, Hans from Qinghai and Tibet were not included, and results can’t represent the current ethnic composition of these minority areas.

In conclusion, our study described the HBV genotypes prevalent in Western China and analyzed the prevalence of antiviral drug resistant mutations in different geographic and ethnic subgroups. The different predominant genotypes in every ethnic population revealed the complex and diversified distribution of HBV genotypes in China. NAs-resistance was found in all of the regions and ethnicities, and Sichuan Han group had more severe resistance than the other minorities. LMV had the highest resistant rate among the four NA agents, while resistance to ETV was the lowest. The influence of HBV genotypes on the development of resistant mutations was weeny, and only more frequent ADV-resistance was found in genotype C patients than genotype B patients in Sichuan Han population. Although HBV genotype detection was not demanded in current guidelines of HBV management, many studies have demonstrated that different genotypes have different clinical features and outcomes. A comprehensive understanding of the molecular epidemiology of HBV will provide evidence for clinical determination. Besides, resistance to NAs could lead to treatment failure and even cross-resistance. The selection of initial therapy and subsequent rescue treatment should be based on the knowledge of resistance rate, and combinations of NAs have been highlighted to refractory patients.

## Methods

### Study design and patients

This was a retrospective study on patients with chronic HBV infection from four areas of Western China: Sichuan, Tibet, Xinjiang and Qinghai. A total of 1316 serum samples from four ethnic groups were recruited, and HBV RT region was successfully amplified from 1221 samples consisting of 639 Sichuan Hans, 18 Xinjiang Hans, 249 Tibet Tibetans, 202 Sichuan Tibetans, 21 Qinghai Tibetans, 46 Xinjiang Uygurs and 46 Sichuan Yis. All the subjects were collected from West China Hospital of Sichuan University, People’s Hospital of Tibet Autonomous region, Tibetan Hospital of Tibet Autonomous region, First Affiliated Hospital of Xinjiang Medical University and People’s Hospital of Qinghai Province from June 2011 to May 2013. All enrolled CHB patients were characterized as HBsAg-positive >6 months, no antibody to hepatitis B surface antigen (anti-HBs) existed, persistent or intermittent elevation of ALT and (or) AST levels, and with no evidence of cirrhosis or carcinoma by imaging and laboratory testing. Patients co-infected with hepatitis A, C, D virus or human immunodeficiency virus (HIV) were excluded. Serum was separated by centrifugation at 4 °C in local hospitals, and stored at −20 °C in West China Hospital of Sichuan University, Chengdu, Sichuan.

Demographic information such as age, ethnicity, family members and residential address were recorded by interview. Since Tibetan patients in Tibet had no family members from other ethnicities, their genetic backgrounds were pure. So did the Uygurs in Xinjiang. This study was approved by the Ethics Review Committee of all participating hospitals, and consistent with the guidelines of the Helsinki Declaration. All participants provided written informed consent for participation in this study.

### HBV DNA quantification and serological tests

Serum HBV viral load was assessed by using real-time fluorescent quantitative polymerase chain reaction (Real Time-PCR) on Roche Light Cycler 480II (Roche Diagnostics, Basel, Switzerland) with a lowest limit of detection of approximately 10^3^ viral genome copies/ml. The handling procedures were carried out in strict accordance with the HBV DNA PCR-Fluorescence Quantitative Diagnostic Kit (Shanghai Kehua Bio-Engineering, Shanghai, China). The reaction volume was 35 μl, and the reaction condition was at 50 °C for 2 min, 94 °C for 5 min, and 40 cycles at 94 °C for 10 s and 60 °C for 45 s, then final extension at 72 °C for 5 min.

Serology markers of HBV including HBsAg, anti-HBs, HBeAg, antibody to hepatitis B e antigen (anti-HBe), and anti-HBc were analyzed using Elecsys Modular E170 immunoassay (Roche Diagnostics, GmbH, Mannheim, Germany) or I2000 immunoassay (Abbott co., USA) in accordance with manufacturers’ instruction.

### HBV DNA extraction and amplification

The HBV nucleic acid was extracted from 200 μl patient serum samples using NucliSENS easyMAG system (Biomerieux Company, Paris, France) following the operators manual. The RT coding region was amplified by designed primers as follows: F: 5′-GTTGCCCGTTTGTCCTC-3′, R: 5′-GACAAACTTTCCAATCAATAGG-3′. A typical amplification was performed in a 20 μl reaction volume containing 1 μl HBV DNA and 0.2 μl Ex Taq HS (TaKaRa Bio, Tokyo, Japan) at 95 °C for 5 min, followed by 40 cycles at 95 °C for 45 sec, 58 °C for 45 sec, and 72 °C for 30 sec, and final extension at 72 °C for 5 min. PCR products were identified using agarose gel electrophoresis and digested by the mixture of shrimp alkaline phosphatase (SAP) and exonuclease I (TaKaRa Bio, Tokyo, Japan) to eliminate the redundant single-strand DNA.

Sample processing, PCR, products identification and digestion was done in separate laboratory rooms which were certified for molecular diagnostics using standard precautions to prevent contamination.

### HBV genotyping and antiviral resistant mutation detection

PCR products of RT region was 529 bp. HBV genotypes and resistant mutations were determined by direct Sanger sequencing the RT region of viral genome on ABI 3130 Genetic Analyzer (Applied Biosystems, South San Francisco, CA, USA).

Sequencing primers was: 5′-ACTTTCCAATCAATAGG-3′. Sequencing reactions were performed in a 5 μl reaction volume containing 1 μl SAP digested PCR products, 0.75 μl BigDye® Terminator v3.1 5xSequencing Buffer, 0.5 μl BigDye® Terminator v3.1 cycle Sequencing RR-100, (Life Technologies/Applied Biosystems, Darmstadt, Germany) and 0.5 μl primers. The sequencing amplification was performed at 96 °C for 1 min, and then 25 cycles at 96 °C for 10 sec, 50 °C for 5 sec, and final extension at 60 °C for 4 min. PCR products were purified by using Bigdye XTerminator Purification Kit (Applied Biosystems, South San Francisco, CA, USA) according to the manufacturer’s instructions.

Purified PCR products were sequencing on ABI 3130 Genetic Analyzer by capillary electrophoresis. The results were analyzed by using software Chromas 2.23 (Technelysium, South Brisbane, QLD, Australia). HBV genotypes were determined according to National Center for Biotechnology Information Genotyping Database (http://www.ncbi.nlm.nih.gov/projects/genotyping/formpage.cgi). Resistant mutations were analyzed by Stanford University HBV RT drug resistance database (http://hivdb.stanford.edu/HBV/DB/cgi-bin/MutPrevByGenotypeRxHBV.cgi)

To confirm the genotyping method, phylogenetic analysis was constructed with the MEGA 5.0 (Tamura, Dudley, Nei, and Kumar 2007), using the Kimura two-parameter matrix model and the Neighbor-Joining method with 1,000 bootstrap replicates. A total of 24 HBV reference sequences were obtained from GenBank, representing nine HBV genotypes A to H. Recombination signals were detected by the SimPlot 3.5.1 (http://sray.med.som.jhmi.edu/SCRoftware/). The bootscan window sizes were 200 bases, the step size was 20 with 100 replicates..

### Statistical Analysis

All statistical analyses were performed using SPSS 13.0 (SPSS Inc., Chicago, IL, USA). Categorical variables, like the percentages of HBV genotypes, were compared by Chi-square test (χ^2^ test) or Fisher’s exact test where cell numbers were small. Mann-Whitney’s U test or Student’s t test was used for continuous variables as appropriate. A two-tailed P < 0.05 was considered to be statistically significant.

## Additional Information

**How to cite this article**: Zhang, Q. *et al.* Epidemiology study of HBV genotypes and antiviral drug resistance in multi-ethnic regions from Western China. *Sci. Rep.* **5**, 17413; doi: 10.1038/srep17413 (2015).

## Supplementary Material

Supplementary Information

## Figures and Tables

**Table 1:** Characteristics of subjects from each ethnicity.

	Han	Tibetan	Uygur	Yi	Total
Total	657	472	46	46	1221
Age	35 (27, 45)	39 (29, 49)	30 (24, 42)	34 (27, 48)	36 (27, 48)
Male (%)	525 (79.8%)	324 (68.64%)	28 (60.87%)	33 (71.73%)	910 (74.53%)
HBV DNA (log10 copies/ml)	5.41 (4.74, 6.99)	6.83 (5.42, 7.15)	7.05 (4.96, 7.56)	6.20 (4.87, 7.04)	6.27 (4.68, 7.05)
HBsAg (%)	657 (100%)	472 (100%)	46 (100%)	46 (100%)	1221 (100%)
Anti-HBs (%)	0	0	0	0	0
HBeAg (%)	645 (98.17%)	453 (95.97%)	46 (100%)	45 (97.82%)	1189 (97.38%)
Anti-HBe (%)	12 (1.83%)	19 (4.03%)	0	1 (2.17%)	32 (2.62%)

Note: Continuous variables were described as median (interquartile range). Table abbreviations: anti-HBc, antibody to the hepatitis B core antigen; anti-HBe, antibody to the hepatitis B e antigen; anti-HBs, antibody to the hepatitis B surface antigen; HBeAg, hepatitis B e antigen; HBsAg, hepatitis B surface antigen; HBV, hepatitis B virus.

**Table 2:** The percentage of mutant sites in different HBV genotype strains in Han population from Sichuan Province.

Mutant site	B (n = 409)		C (n = 230)		P value
	Number	Percentage (%)	Number	Percentage (%)	
rtV173L	3	0.7	4	1.7	0.259^a^
rtL180M	62	15.2	48	20.9	0.066^b^
rtA181V/T/S	21	5.1	31	13.5	0.000^b^,*
rtT184A/I/S	5	1.2	5	2.2	0.508^a^
rtT184L	3	0.7	1	0.4	1.000^a^
rtS202G	9	2.2	1	0.4	0.104^a^
rtM204V	52	12.7	23	10.0	0.306^b^
rtM204I	96	23.5	50	21.7	0.617^b^
rtM204V/I	5	1.2	5	2.2	0.508^a^
rtV207M/I/L	10	2.4	8	3.5	0.449^b^
rtS213T	9	2.2	5	2.2	0.982^b^
rtV214A	0	0	5	2.2	0.006^a^,*
rtN236T	26	6.4	12	5.2	0.559^b^
rtP237H	0	0	6	2.6	0.002^a^,*
rtN/H238T	0	0	12	5.2	0.000^a^,*
rtM250L	2	0.5	0	0.0	0.539^a^

Note: Percentage = number of single mutant site of genotype B(C)/number of genotype B(C); *statistically significant.

^a^Fisher’s exact test, two-sided.

^b^χ^2^ test, two-sided.

**Table 3:** The percentage of drug resistant mutation models in different HBV genotype strains in Han population from Sichuan Province.

Characteristics	B (n = 192)		C (n = 112)		P value
	Number	Percentage (%)	Number	Percentage (%)	
rtL180M+rtM204V	28	14.6	10	8.9	0.150^a^
rtL180M+rtM204I	8	4.2	12	10.7	0.026^a^,*
rtL180M+rtM204V/I	2	1.0	4	3.6	0.198^b^
rtL180M+rtM204V+rtV173L	1	0.5	4	3.6	0.063^b^
rtA181T+rtN236T	5	2.4	3	2.7	1.000^b^
rtA181T+rtM204I	2	1.0	1	0.9	1.000^b^
rtL180M+rtM204V+rtS202G	6	3.1	1	0.9	0.267^b^
rtL180M+rtM204V+rtT184L	3	1.6	1	0.9	1.000^b^
rtL180M+rtM204V+rtM250L	1	0.5	0	0	1.000^b^

Note: Percentage = number of mutant model of genotype B(C)/number of genotype B(C);

*statistically significant.

^a^χ^2^ test, two-sided.

^b^Fisher’s exact test, two-sided.
