# Corrigendum: Epidemiology study of HBV genotypes and antiviral drug resistance in multi-ethnic regions from Western China

**DOI:** 10.1038/srep20451

**Published:** 2016-01-29

**Authors:** Qi Zhang, Yun Liao, Jie Chen, Bei Cai, Zhenzhen Su, Binwu Ying, Xiaojun Lu, Chuanmin Tao, Lanlan Wang

Scientific Reports 5: Article number: 1741310.1038/srep17413; published online: 11272015; updated: 01292016

The Acknowledgements section in this Article is incomplete.

“This study was supported by National Natural Science Foundation of China (Grant No. 81273256, Grant No. 81301507, and Grant No. 81401666).”

should read:

“We thank Min Zhao (Department of Laboratory Medicine, People’s Hospital of Tibet Autonomous region, Lhasa 850000, China), Zhu Ba (Department of Laboratory Medicine, Tibetan Hospital of Tibet Autonomous region, Lhasa 850000, China), Zhaoxia Zhang (Center of Laboratory Medicine, First Affiliated Hospital of Xinjiang Medical University, Urumchi 830054, China), and Xinbo Song (Department of Laboratory Medicine, West China Hospital, Sichuan University, Chengdu 610041, China) for helpful sample collection.

This study was supported by National Natural Science Foundation of China (Grant No. 81273256, Grant No. 81301507, and Grant No. 81401666).”

