# Supplementary material for: Epidemiology study of HBV genotypes and antiviral drug resistance in multi-ethnic regions from Western China
Source: Sci Rep. 2015 Nov 27;5:17413. doi: 10.1038/srep17413 (PMC4661727; doi:10.1038/srep17413)
Supplement: Supplementary Information [file srep17413-s1.pdf]

Epidemiology study of HBV genotypes and antiviral drug resistance in multi-ethnic regions from Western China

Qi Zhang#, Yun Liao#, Jie Chen, Bei Cai, Zhenzhen Su, Binwu Ying, Xiaojun Lu, Chuanmin Tao, Lanlan Wang*

Department of Laboratory Medicine, West China Hospital, Sichuan University, Chengdu, 610041, China

*Corresponding Author: Lanlan Wang
Department of Laboratory Medicine, West China Hospital, Sichuan University, No.37 Guoxue Alley, Wuhou district, Chengdu 610041, People's Republic of China
E-mail: lanlanwang2014@126.com
Tel: 86-28-85422751; Fax: 86-28-85422751

#, Qi Zhang and Yun Liao contributed equally to this work

Supplementary legends

Supplementary Table S1. HBV genotypes and clinical characteristics in Han population

Supplementary Table S2. Geographic and demographic distribution of HBV antiviral resistant rate in Western China

Supplementary Table S3. HBV genotypes and antiviral resistance in Han population

Supplementary Table S4. The prevalence of mutations in different HBV genotypes in lamivudine-resistance Sichuan Han patients

Supplementary Table S5. The prevalence of mutations in different HBV genotypes in telbivudine-resistant Sichuan Han patients

Supplementary Table S6. The prevalence of mutations in different HBV genotypes in adefovir-resistant Sichuan Han patients

Supplementary Table S7. The prevalence of mutations in different HBV genotypes in entecavir-resistant Sichuan Han patients

Supplementary Table S1. HBV genotypes and clinical characteristics in Han population

	genotype B	genotype C	P value
n	409	230	
Age	34.90±10.218	37.34±10.334	0.004[a,]*
Male % (n/N)	80.4% (329/409)	80.4% (185/230)	0.999[b]
HBV-DNA (log10 copies/ml)	5.43±1.49	5.21±1.52	0.25[a]
TB (μmol/L)	40.62±67.35	43.37±77.68	0.76[a]
DB (μmol/L)	24.86±51.49	26.00±55.74	0.86[a]
ALT (IU/L)	222.00±437.41	180.48±325.65	0.40[a]
AST (IU/L)	152.58±337.50	136.58±270.38	0.68[a]
ALB (g/L)	43.19±7.51	43.52±6.34	0.71[a]
GLB (g/L)	30.00±5.58	30.70±5.10	0.29[a]
ALP (IU/L)	94.73±45.29	104.15±53.63	0.12[a]
GGT (IU/L)	53.51±65.05	64.27±75.02	0.21[a]

Note: *, statistically significant; Continuous variables were described as mean±SD.

Table abbreviations: TB, total bilirubin; DB, direct bilirubin; ALT, alanine aminotransferase; AST, aspartame aminotransferase; ALB, albumin; GLB, globulin; ALP, alkaline phosphatase; GGT, gamma glutamyl transpeptidase

[a]Student's *t* test, two-sided.

[b]χ^2 test, two-sided.

Supplementary Table S2. Geographic and demographic distribution of HBV antiviral resistant rate in Western China

Ethnic population	LMV	LdT	ADV	ETV	Total
Sichuan Hans	43.5%	38.1%	13.4%	4.2%	47.5%
Sichuan Yis	17.4%	17.4%	6.5%	0.0%	23.9%
Qinghai Tibetans	14.3%	4.8%	4.8%	0.0%	19.0%
Xinjiang Hans	16.7%	0.0%	0.0%	0.0%	16.7%
Xinjiang Uygurs	8.7%	2.2%	0.0%	0.0%	8.7%
Tibet Tibetans	4.4%	0.0%	0.0%	0.0%	4.4%
Sichuan Tibetans	3.2%	0.0%	0.0%	0.0%	3.2%

Note: LMV, lamivudine; LdT, telbivudine; ADV, adefovir; ETV, entecavir.

Supplementary Table S3. HBV genotypes and antiviral resistance in Han population

	LMV resistance n (%)	LdT resistance n (%)	ADV resistance n (%)	ETV resistance n (%)	Overall resistant rate n (%)
B (n=409)	172(42.1%)	153(37.4%)	39(9.5%)	19(4.7%)	192(46.9%)
C (n=230)	106(46.1%)	91(39.6%)	46(20.0%)	8(3.5%)	112(48.7%)
P value[a]	0.324	0.590	0.000*	0.481	0.670

Note: *, statistically significant; LMV, lamivudine; LdT, telbivudine; ADV, adefovir; ETV, entecavir.

[a]χ^2 test, two-sided.

Supplementary Table S4. The prevalence of mutations in different HBV genotypes in lamivudine-resistance Sichuan Han patients

	rtV173L N(%)	rtL180M N(%)	rtA181V/T/S N(%)	rtM204V N(%)	rtM204I N(%)	rtV207M/I/L N(%)	rtS213T N(%)
B(N=172)	3(1.7%)	62(36.1%)	18(10.5%)	52(30.2%)	96(55.8%)	10(5.8%)	9(5.2%)
C(N=106)	4(3.8%)	58(54.7%)	31(29.3%)	23(21.7%)	50(47.2%)	8(7.6%)	5(4.7%)
p value	0.433[a]	0.002[b,]*	0.000[b,] *	0.119[b]	0.161[b]	0.568[b]	0.849[b]

Note: *, statistically significant.

[a]Fisher’s exact test, two-sided.

[b]χ^2 test, two-sided.

Supplementary Table S5. The prevalence of mutations in different HBV genotypes in telbivudine-resistant Sichuan Han patients

	rtV173L N(%)	rtL180M N(%)	rtT184A/I/S N(%)	rtS202G N(%)	rtM204V N(%)	rtM204I N(%)	rtM250L N(%)
B(N=153)	3(1.9%)	62(40.5%)	5(3.3%)	9(5.9%)	48(31.4%)	96(62.8%)	2(1.3%)
C(N=91)	4(4.4%)	58(63.7%)	5(5.5%)	1(1.1%)	22(24.2%)	50(54.9%)	0(0.0%)
P value	0.429[a]	0.000[b,]*	0.507[a]	0.096[b]	0.229[b]	0.229[b]	0.530[a]

Note: *, statistically significant.

[a]Fisher’s exact test, two-sided.

[b]χ^2 test, two-sided.

Supplementary Table S6. The prevalence of mutations in different HBV genotypes in adefovir-resistant Sichuan Han patients

	rtA181V/T/S N(%)	rtN236T N(%)
B(N=39)	21(53.9%)	26(66.7%)
C(N=46)	18(39.1%)	6(13.0%)
P value[a]	0.175	0.000*

Note: *, statistically significant.

[a]χ^2 test, two-sided.

Supplementary Table S7. The prevalence of mutations in different HBV genotypes in entecavir-resistant Sichuan Han patients

	rtL180M N(%)	rtT184A/I/S/L N(%)	rtS202G N(%)	rtM204V N(%)	rtM204I N(%)	rtM204V/I N(%)	rtM250L N(%)
B(N=18)	17(94.4%)	8(44.4%)	9(50.0%)	15(83.3%)	2(11.1%)	1(5.6%)	2(11.1%)
C(N=6)	6(100%)	5(83.3%)	1(16.7%)	4(66.7%)	1(16.7%)	1(16.7%)	0 (0.0%)
P value[a]	1.000	0.166	0.341	0.394	1.000	0.446	1.000

Note: [a]Fisher's exact test, two-sided.
